# Heparanase inhibitors restrain mesothelioma

**DOI:** 10.18632/oncotarget.26243

**Published:** 2018-12-07

**Authors:** Moshe Lapidot, Uri Barash, Israel Vlodavsky, Harvey Pass

**Affiliations:** ^1^ Cancer and Vascular Biology Research Center, Rappaport Faculty of Medicine, Technion, Haifa, Israel; ^2^ Department of Cardiothoracic Surgery, Langone Medical Center, New York University School of Medicine, New York, USA

**Keywords:** mesothelioma, heparanase, PG545, defibrotide, therapies

## Abstract

Malignant mesothelioma is a highly aggressive form of cancer with poor prognosis due to lack of markers for early diagnosis and resistance to conventional therapies. Heparanase, the sole heparan sulfate (HS) degrading endoglycosidase, regulates multiple biological activities that enhance tumor growth, metastasis, angiogenesis, and inflammation. Heparanase accomplishes this by degrading HS and thereby facilitating cell invasion and regulating the bioavailability of heparin-binding proteins. Applying pre-clinical and clinical models of human mesothelioma and potent inhibitors of heparanase enzymatic activity (PG545, Defibrotide) we investigated the significance of heparanase in the pathogenesis of mesothelioma. We found that mesothelioma tumor growth was markedly attenuated by heparanase gene silencing and by heparanase inhibitors. Furthermore, heparanase inhibitors were more potent *in vivo* than conventional chemotherapy. Clinically, heparanase levels in patients’ pleural effusions could distinguish between malignant and benign effusions, and heparanase H-score (immunostaining of tumor specimens) above 90 was associated with reduced patient survival. These results strongly imply that heparanase plays an important role in mesothelioma tumor progression, thus encouraging the use of heparanase inhibitors in combination with existing drugs as a new therapeutic modality in mesothelioma clinical trials.

## HIGHLIGHTS

**Figure 1:**
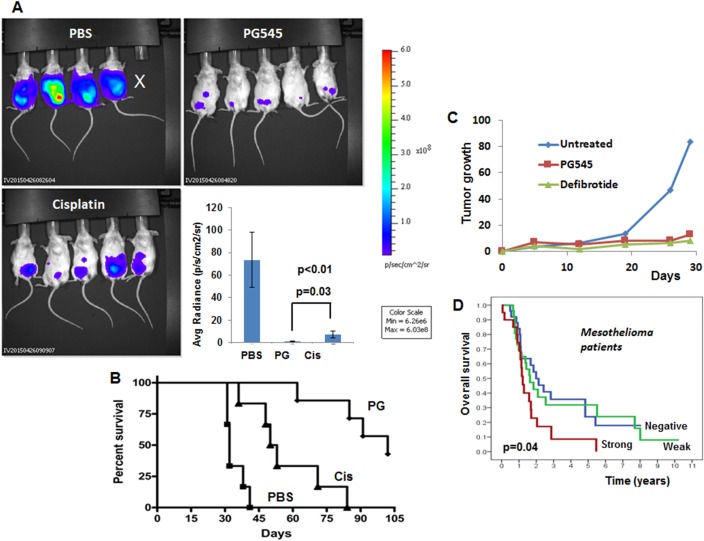
Heparanase a valid target in the pathogenesis of mesothelioma **A.** Tumor growth. Luciferase-labeled MSTO-211H human mesothelioma cells (2×10^6^) were inoculated i.p into SCID mice. Mice were treated with PG545 (400 mg/mouse; once a week), cisplatin (once/2 weeks; 3 mg/kg), or control vehicle (PBS) and tumor development was inspected by IVIS. Quantification of the luciferase intensities is shown graphically in the lower right panel. **B.** Survival. The effect of PG545 and cisplatin on the survival of mice is plotted as Kaplan–Meier curves. Note that PG545 is significantly more effective than cisplatin in inhibiting orthotopic mesothelioma tumor growth and prolonged survival of the mice. **C.** Defibrotide. Luciferase-labeled MSTO-211H human mesothelioma cells (0.5×10^6^) were inoculated i.p into SCID mice. Mice were treated with defibrotide (8 mg/mouse; twice a day), PG545 (400 mg/mouse; once a week) or control vehicle (PBS) and tumor development was inspected by IVIS over time. **D.** Kaplan–Meier survival analysis of patients according to their heparanase staining intensities. Mesothelioma patients endowed with strong heparanase staining survive less than patients that are found negative for heparanase (*p* = 0.04 for strong *vs*. negative).

Malignant pleural mesothelioma (MPM), the most common form of mesothelioma, is a highly aggressive tumor characterized by rapid and diffused local growth in the thoracic cavity. Multiple observations support a scenario whereby mesothelial cells undergo a series of chronic injury, inflammation, and proliferation during the long latency period of malignant mesothelioma that is perpetuated by durable fibers, the tumor microenvironment, and inflammatory stimuli [1, 2]. MPM has a poor prognosis because of the lack of markers for early diagnosis and resistance to conventional therapies. Surgery, radiotherapy, and chemotherapy have failed as single modality therapies and the first-line standard chemotherapy of MPM is the combination of cisplatin and pemetrexed. This treatment regimen confers a median progression-free survival of 5.7 months; no alternative can be offered when this treatment fails, thus underscoring the urgent needs for novel treatments modalities. A recent publication in the Journal of the National Cancer Institute (JNCI) elucidated a novel molecular pathway that promotes mesothelioma growth by the enzyme heparanase [3]. Unlike other classes of extracellular matrix (ECM) degrading enzymes (i.e., matrix metalloproteinases, cathepsins) mammalian cells express a single functional heparanase, an endoglycosidase that cleaves heparan sulfate (HS) side chains of HS proteoglycans. Cleavage of HS by heparanase leads to disassembly of the ECM, thereby promoting cell dissemination associated with tumor metastasis, angiogenesis and inflammation [4, 5]. Heparanase is up-regulated in essentially all human tumors examined [6, 7]. Notably, cancer patients exhibiting high levels of heparanase had a significantly shorter postoperative survival time than patients whose tumors exhibit low levels of heparanase [4, 5]. Compelling evidence ties heparanase levels with all steps of tumor formation including tumor initiation, growth, metastasis, and chemoresistance [6, 7]. These and other results indicate that heparanase is causally involved in cancer progression and hence is a valid target for anti-cancer drug development. This notion is reinforced by preclinical studies revealing a marked inhibition of tumor growth in mice treated with heparanase-inhibitors (i.e., roneparstat, pixatimod) [6, 7] and neutralizing antibodies [8]. Importantly, heparanase mediates, through enzymatic and non-enzymatic activities, a vicious pro-inflammatory and pro-tumorigenic crosstalk in the tumor microenvironment by regulating gene expression, remodeling the ECM, and facilitating growth factor bioavailability and signaling [9], all highly relevant to mesothelioma tumorigenicity and chemoresistance [1–3]. It appears that heparanase secreted by tumor and/or host cells primes the tumor microenvironment to better support mesothelioma tumor growth and drug resistance. Applying extensive pre-clinical mouse models, Barash, Lapidot et al [3] show that mesothelioma tumor growth is markedly attenuated by heparanase-inhibiting compounds (Figure 1), heparanase gene silencing, and by inoculation of mesothelioma cells into heparanase null mice. Clinically, patients with high heparanase immunostaining or H-score survived less than patients with low levels of heparanase (Figure 1D). Even a higher survival rate is obtained in a sub-group of patients in which heparanase H-score was calculated before chemotherapy. This may suggest that chemotherapy induces heparanase expression, thus urging the addition of heparanase inhibitors to conventional chemotherapy. In addition, we found that heparanase levels (ELISA) can provide valuable information and categorize the plasma/effusion sample as malignant or benign with high confidence, implying its diagnostic potential. The clinical results are supported by the ability of heparanase inhibitors to prominently restrain the growth of mesothelioma tumor xenografts implanted orthotopically (Figure 1A). Notably, the heparanase inhibitor PG545 (= pixatimod, a highly sulfated tetra-saccharide bound to a lipophilic cholestanol aglycone) appeared more effective than cisplatin, a common chemotherapeutics in mesothelioma, in restraining tumor growth, closely associating with a profoundly prolonged survival of mesothelioma-bearing mice (Figure 1B). Similar potency was also noted for the heparanase inhibitor defibrotide (= Defitelio; a polydisperse oligonucleotide found to inhibit heparanase gene expression and enzymatic activity) (Figure 1C). Since defibrotide is an approved drug for vascular occlusion disease (VOD), its effectiveness in oncology may bring heparanase inhibitors closer to the clinic. Further studies are needed, but this new class of inhibitors may bring hope to mesothelioma patients. Altogether, it appears that heparanase is a master regulator of the aggressive phenotype of malignant mesothelioma, an important contributor to the poor outcome of mesothelioma patients and a prime target for therapy, encouraging clinical examination of heparanase inhibitors as a new therapeutic modality in mesothelioma.
